# Interactions between CO and Poly(p-phenylene vinylene) as Induced by Ion-Exchanged Zeolites

**DOI:** 10.3390/ma2042259

**Published:** 2009-12-08

**Authors:** Nareerat Thongchai, Ruksapong Kunanuruksapong, Sumonman Niamlang, Ladawan Wannatong, Anuvat Sirivat, Sujitra Wongkasemjit

**Affiliations:** 1 Conductive and Electroactive Polymers Research Unit, and Center of Petroleum Petrochemicals and Advanced Materials, The Petroleum and Petrochemical College, Chulalongkorn University, Bangkok 10330, Thailand; E-Mails: nareerat_thongchai@yahoo.com (N.T.); amd64best@hotmail.com (R.K.); tanggggo@hotmail.com (S.N.); sujitra.d@chula.ac.th (S.W.); 2 Department of Materials and Production Technology Engineering, King Mongkut’s University of Technology, Bangkok 10800, Thailand; E-Mail: ruangchuay@gmail.com (L.W.)

**Keywords:** conductive polymer, electrical conductivity, CO, ion-exchanged zeolites

## Abstract

The effects of zeolite type, ion-exchanged level, and ion type on the electrical conductivity responses of poly(*p*-phenylene vinylene) (PPV), doped poly(*p*-phenylene vinylene) (dPPV) and zeolite composites under CO exposures were investigated. The electrical sensitivity of dPPV/Cu^+^-ZSM5(Si/Al = 23) system shows a negative sensitivity value of −0.154, while the Na^+^ system gives a positive sensitivity of 1.48. Based on FTIR and TPD data, the positive response of PPV/Na^+^-ZSM5 stems from the CO molecules acting as a secondary dopant. On the other hand, the negative response of PPV/Cu^+^-ZSM5 originates from the fact that CO molecules are selectively adsorbed on Cu^+^ sites rather than C^+^ sites of doped PPV.

## 1. Introduction

Energy from fossil fuels is associated with the production of toxic by-products such as CO, NO_x_, and hydrocarbons. A significant part of CO and NO_x_ emissions originate from motor vehicles. The interaction of CO and NO_x_, in which sunlight tends to produce O_3_ with the resulting strongly oxidizing behavior, is believed to be harmful to plants and to the respiratory system of human beings [1,2]. Moreover, toxic air contaminants may accumulate in the body from long-term exposure and contributes to a wide range of diseases. Carbon monoxide (CO) can cause chest pains in heart patients, headaches, nausea and reduced mental alertness [2].

Conducting polymers such as poly(*p*-phenylene vinylene) (PPV) is a candidate as an active sensing material in devices because PPV possesses good optical and electrical properties, and it can be synthesized by a relative simple technique [3,4,5,6,7,8,9,10,11,12,13,14]. To obtain highly effective sensors, selected materials should have very narrow chemical specificity along with high sensitivity towards polar chemicals. A zeolite is chosen as a selective microporous adsorbent to be introduced into a polymer matrix in order to alter to sensitivity towards a particular gas such as CO.

Carbon monoxide is a widely employed as a probe molecule in surface chemistry to titrate the number and the strength of acidic sites by comparing shift and intensity of the vibrational mode of adsorbed CO to gas-phase molecules. CO molecules interact with Lewis acid centers when exposed to a surface of cations of metal oxides, halides, and with cations of metal-exchanged zeolites. The interaction of CO with the surface of ionic oxides and halides has been extensively investigated theoretically [15,16,17,18,19]. These results provide a solid basis for the understanding of the bond nature of the probe molecule to the acid sites and of the origin of the CO frequency shift. The interaction of CO with a transition metal cation implies the donation of electronic charge from the 5p orbital of CO to a *d* orbital of the metal in synergy with a back-donation from the metal to the empty anti-bonding 2π* orbital of CO. In the case of cations without *d* orbtals in their valence shell no such “chemical” bonding can occur and the interaction is essentially mediated by electrostratics [20,21,22,23].

In the past, several studies have been devoted to the interaction of CO with metal-exchanged zeolites by using Fourier transform infrared technique [24,25,26,27,28,29,30,31,32,33]. In our work, we have systematically investigated the interactions between CO with poly(p-phenylene vinylene), Na^+^-exchanged zeolite, Cu^+^-exchange zeolite, and the poly(p-phenylene vinylene)/zeolite composites with a view to possible gas sensing applications.

## 2. Experimental Methods

α,α’-Dichloro-*p*-xylene (Aldrich) and tetrahydrothiophene (Aldrich) were used to synthesize PPV monomers. Analytical grade methanol (Merck) was used as the solvent. Sodium hydroxide (Merck) and hydrochloric acid (Merck) were used as the basic and the acid reagents, respectively. Sulfuric acid (Merck) was used as the oxidant. Sodium chloride (Merck) and copper(II) chloride (Merck) were used as the ion-exchanged agents. Zeolite: Na-13X (Aldrich), NH_4_-ZSM5 (Si/Al = 23, 80) (Zeolyst), NH_4_-Ferrierite (Zeolyst) were used as the adsorbents. All chemicals were used without further purification. Carbon monoxide (2,000 ppm in nitrogen, TIG) was used as the target gas. Nitrogen (N_2_, TIG) was used as the surface cleaning gas.

Twenty scans of a FT-IR spectrometer (Bruker, model FRA 106/S) with a resolution of 4 cm^–1^ were used to characterize functional groups and the frequency changes of the samples. To characterize the frequency changes of CO when interact with dPPV or zeolite, an IR spectrum of pure doped PPV or zeolite is subtracted from an IR spectrum of CO adsorbed on doped PPV or zeolite. A thermogravimetric analyzer (Dupont, model TGA 2950) with a heating rate 10 °C/min under N_2_ atmosphere was used to characterize the PPV precursor, PPV, and the doped PPV. An X-ray diffractometer (Rigaku, model D/MAX-2000) was used to determine the degrees of crystallinity of PPV and the doped PPV, and the crystal order of the zeolites. A scanning electron microscope (SEM, JEOL, model JSM-5200) was used to study the morphology of PPV, the doped PPV, the zeolites, and the PPV/Zeolite composites at the magnifications of 1,500 and 5,000 and at 15 kV. The BET (Sorptomatic-1990) was used to measure the pore sizes and the surface areas of the zeolites. An X-ray fluorescence spectrometer (Bruker, model SRS 3400) was used to measure the Cu^+^ and Na^+^ exchanged capacities of the zeolites. An electron spin resonance (ESR) spectrometer (Varian E-109) was used to observe an oxidation state of cupper. The TPD method was applied to the analysis of the CO adsorbed species on Na^+^ZSM5 and Cu^+^ZSM5 samples by using He as the carrier gas at the rate of 60 cm^3^ min^–1^, and the heating rate of 5 K·min^–1^. A custom made two-point probe with a linear geometric array was used to measure the specific conductivity.

Poly(*p*-phenylene vinylene) in our work was synthesized by the sulfonium precursor route [2]. Synthesis of the *p*-xylene-bis(tetrahydrothiophenium chloride) monomer was achieved by reacting α,α’-dichloro-*p*-xylene at a concentration of 0.75 M with excess tetrahydrothiophene at 50 °C in methanol: water (80:20) solution for 24 h. This monomer was purified in the reaction solution, followed by precipitating the product in cold acetone (0 °C), filtration, and vacuum drying. The precursor sulfonium polyelectrolyte, poly[*p*-xylene-bis(tetrahydrothiophenium chloride)], was prepared in an aqueous solution by the base induced polymerization of an appropriate *bis*-sulfonium monomer. The reaction was usually carried out at low temperatures in fairly dilute monomer solutions and in equimolar base to monomer ratios in order to suppress the premature formation of unsaturated polymer segments by thermal or base induced elimination of solubilizing side chains. The polymerization reaction was terminated by the addition of dilute aqueous hydrochloric acid to the reaction mixture which was then dialyzed against water in order to separate the high molecular weight fraction from the monomeric and oligomeric residues and the sodium and chloride ions. Poly(p-phenylene vinylene) was essentially obtained by heating poly(p-xylene-bis(tetrahydrothiophenium chloride)) under vacuum at 180 °C for 6h. PPV particles were subsequently doped with sulfuric acid with the mole ratio N_PPV_:N_H2SO4_ equal to 1:10 at [H_2_SO_4_] = 9 M.

Zeolites Na^+^-13X , NH_4_^+^-ZSM5 (Si/Al = 23, 80), and NH_4_^+^-ferrierite were calcined at 473 K for 2 hours prior to their use. Na^+^ contained zeolites, ZSM5(Si/Al = 23, 80), and ferrierite were prepared by immersing 1 g of zeolite powder in 100 ml of 0.3M NaCl for 12 hours at room temperature. The precipitate was then filtered, washed twice with hot water, and then dried at 353 K for 2 hours. Cu^+^ ion-exchanged zeolite was obtained by stirring the zeolite sample in 0.3 M CuCl_2_ solution at room temperature for 12 hours and activated under vacuum at 573 K for 2 hours [6,7]. PPV/zeolite composites were prepared by dry mixing PPV particles with the zeolites at the volume ratio equal to 1:10 [8]. The composites were compressed into a disc form by a hydraulic press at pressure equal to 6 kN and the conductivity values were measured by the two-point probe technique.

The specific conductivity σ (S/cm) values of the pellets were obtained by measuring the bulk pellet resistance R (W). The relation σ = (1/R*t*)(1/K) was used to calculate specific conductivity, where *t* is the pellet thickness and K is the geometric correction factor which is equal to the ratio *w/l*, where *w* and *l* are the probe width and the length, respectively. K value was determined by calibrating the four-point probe with semiconducting silicon sheets of known resistivity values. Electrical conductivity values of several samples were first measured at various applied dc currents to identify their linear Ohmic regimes.

A custom-built gas detection unit was made consisting of two chambers of equal volume: a mixing chamber is connected in series with a working chamber. Temperature and pressure in both chambers were controlled and monitored. The operating temperature was fixed at 30 ± 5 °C during the experiment. The procedure was as follows. Initially, the steady state electrical conductivity of sample in air was measured and then both chambers were evacuated until the sample conductivity decreased to a nearly constant value; presumably the moisture content was reduced to its minimum value. Then N_2_ at 1 atm and 30 ± 2 °C was injected into the working chamber and the electrical conductivity value was recorded. The procedure of evacuation and N_2_ injection was repeated again and again until the steady state conductivity in N_2_ (σ_N2,before-expose_) was close to the electrical conductivity value in vacuum. Then N_2_ was then evacuated from the working chamber. CO was injected into the mixing chamber until the pressure reached 2 atm and at 30 °C. Half of the gas mixture was then allowed to escape into the working chamber where now the pressures in both chambers were reduced to 1 atm. The conductivity value was recorded through a source meter (Keithley, model 6517A) connected to a PC. After the steady state conductivity value was obtained and recorded, CO was evacuated. N_2_ was injected into the working chamber until its pressure reached 1 atm. The electrical conductivity after exposing CO was recorded (σ_N2,after-expose_). The electrical conductivity response, Δσ, to CO was calculated from the equation: Δσ = σ_CO_ − σ_N2_,_before-expose_ where σ_CO_ is the steady state electrical conductivity value (S/cm). A small difference of few percents was observed between σ_N2,after-expose_ and σ_N2, before-expose_.

The sample for IR measurements was prepared as a self supporting wafer and was placed in a cell at room temperature with gas in situ. The procedure allows IR spectra to be measured during adsorption process of the probe molecule. The spectra were recorded at room temperature by a spectrophotometer (Bruker, model FRA 106/S) with a resolution of 4 cm^–1^. The spectrum of adsorbed species was obtained by subtracting a spectrum of the gas-phase CO from the background one.

## 3. Results and Discussion

### 3.1. Characterization of Poly(p-phenylene vinylene)

FTIR spectroscopy data of PPV precursor, PPV, and sulfuric acid doped PPV are tabulated in Table 1. For PPV precursor, the presence of the absorption band near 960 cm^–1^ occurs from the C-H out-of-plane bending, indicating a characteristic of the trans configuration of the vinylene group [32,33]. The absorption band at around 3,022 cm^–1^ is due to the *trans* vinylene C-H stretching mode. The absorption band at around 550 cm^–1^ is attributed to the phenylene out-of-plane ring-bending. The bands at 830 cm^–1^ and 1,511 cm^–1^ can be assigned to the *para*-phenylene ring C-H out-of-plane bending and the C-C ring stretching, respectively. The bands at 2,872 and 2,960 cm^−1^ can be attributed to the CH_3_ symmetric and CH_3_ asymmetric deformation [32,33]. After the heat treatment under vacuum, the intensities of these two bands decrease. The intensity of the absorption band near 3,022 cm^–1^ increases due to the elimination of the tetrahydrothiophenyl group and HCl. The absence of the C-S linkage peak at 632 cm^–1^ from tetrahydrothiophene indicates the full conversion of the precursor after pyrolysis [34]. Upon oxidation of PPV, the infrared spectrum shows new bands at 1,550, 1,485, 1,316, 1,280, 1,150, and 876 cm^–1^. The emergence of these new bands in the spectra is related to the formation of the quinoid structures [34]. The quinoid structure is a result of a symmetry breaking of the polymeric chain. Although formation of the quinoid structure is evident for the doping agent used, some bands associated with benzoid structure (undoped PPV) survive after doping the polymer. Therefore, even for extensive oxidation, only a partial oxidation of the polymer takes place and the two structures coexist.

**Table 1:** Peak positions from FT-IR spectra of PPV precursor, PPV, and doped PPV with a mole ratio of sulfuric acid to monomer unit equal to 10:1.

Functional groups	Wavenumber (cm^–1^)			References
	PPV Precursor	PPV	doped PPV	
Phenylene out of plane ring bending	560	557	557	[32]
	[550 ± 10]	[550 ± 10]	[550 ± 10]	
C-S stretching	637	_	_	[33]
	[632 ± 10]			
S-O stretching	_	_	640	[33]
			[650 ± 10]	
*para*-phenylene ring C-H out of plane bending	840	837	837	[32]
	[830 ± 10]	[830 ± 10]	[830 ± 10]	
C-H out of plane bending	950	963	963	[32]
	[960 ± 10]	[960 ± 10]	[960 ± 10]	
S=O symmetric stretching	_	_	1,056	[33]
			[1,050 ± 10]	
Quinoid ring C=C stretching	_	_	1,177	[33]
			[1,170 ± 10]	
S=O asymmetric stretching	_	_	1,204	[33]
			[1,200 ± 10]	
C-C ring stretching	1,513	1,517	1,517	[32]
	[1,517 ± 10]	[1,517 ± 10]	[1,517 ± 10]	
CH_3_ symmetric stretching	2,880	2,882	2,880	[32]
	[2,872 ± 10]	[2,872 ± 10]	[2,872 ± 10]	
CH_3_ asymmetric stretching	2,950	2,961	2,962	[32]
	[2,960 ± 10]	[2,960 ± 10]	[2,960 ± 10]	
*trans*-vinylene C-H strectching	_	3,023	3,023	[32]
		[3,022 ± 10]	[3,022 ± 10]	

* peak positions in blanket cited from references.

**Figure 1:**
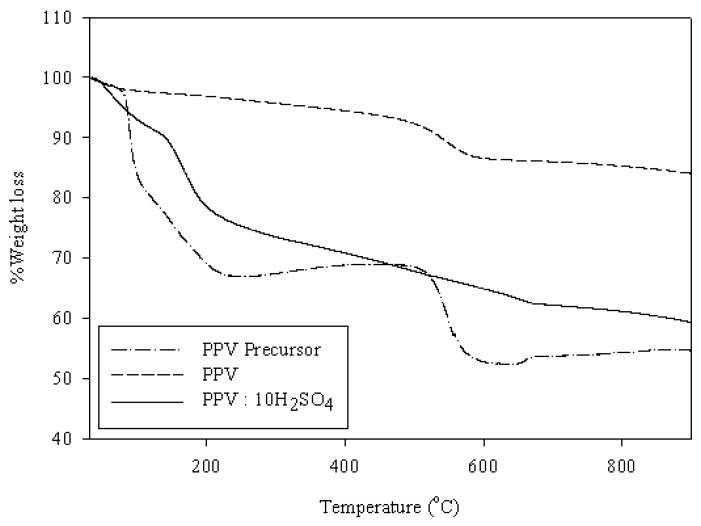
TGA thermogram of PPV Precursor, PPV and doped PPV.

The thermal behaviors of the polymers are shown in Figure 1. There are three transitions for the PPV precursor around 80–150 °C, 150–230 °C and 520–580 °C. The first one is due to the removal of the solvent from the polymer. The second transition, around 150–230 °C, is related to the elimination reaction, yielding tetrahydrothiophene and HCl, which converts the PPV precursor to PPV. The third transition is attributed to the degradation reaction of the main chain [33]. After the pyrolysis of the PPV precursor under vacuum for 6 h, the thermogram shows two transitions. The weight loss around 50–80 °C is due to the removal of the physically absorbed water. The transition temperature occurring at around 450–580 °C can be attributed to the decomposition of polymer [8]. Therefore, the TGA results confirm that tetrahydrothiophene and HCl were eliminated from the PPV precursor after the thermal treatment. The thermal behavior of sulfuric acid doped PPV shows three step weight loss. The weight loss around 50–105 °C is attributed to the diffusion of physisorbed water. The second step, 50–250 °C, is due to the loss of counterion belonging the dopant. The last step, 600–680 °C, is related to the degradation of polymer [33]. The TGA results of PPV and doped PPV show that the doped PPV has higher thermal stability; after doping the degradation temperature of the sulfuric acid doped PPV is higher than that of the pristine PPV.

From XRD patterns, the interplanar spacing between aromatic group can be identified with the peak at 24.38 Å for PPV. After chemically oxidized with H_2_SO_4_, the doped PPV possesses peaks at 20.58 Å and 24.28 Å, which can be related to the layer of the dopant and the polymer chain, respectively. The doping process does not disrupt the original orientation of the PPV crystallites and the crystalline phase obtained with the dopant [35]. Based on this evidence, an electrically conductive phase consisting of layers of polymers separated by a layer of the chemical dopant can be proposed. The particle size analysis gives the particle diameters of PPV and the doped PPV of about 52.97 and 62.72 µm, respectively. The standard deviations are 6.97 and 0.84 µm, respectively. This is due to the introduction of charges upon doping, inducing PPV particles to agglomerate on the micro scale from electrostatic interaction. The apparent densities of PPV and doped PPV are about 0.840 g/cm^3^ with standard deviations of 0.13 and 0.09 g/cm^3^, respectively.

### 3.2. Characterization of Zeolite

**Figure 2:**
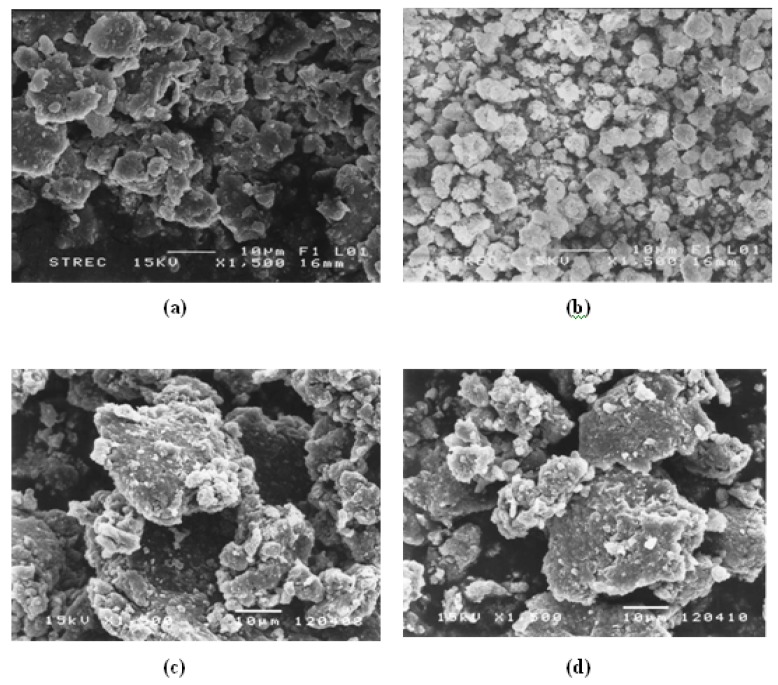
SEM micrographs of powder samples at the magnification 1,500 of: (a) doped PPV; (b) NH_4_^+^-ZSM5 (Si/Al = 23) before the ion-exchanges; (c) doped PPV/Na^+^-ZSM5; and (d) doped PPV/Cu^+^-ZSM5.

The XRD patterns of zeolites: 13X, ZSM5 (Si/Al = 23, 80), and Ferrierrite covering angles between 2q = 5–90° were measured. The major peaks of the zeolites are consistent with those in previously published work [36]. The BET specific surface areas of 13X, ZSM5 (Si/Al = 23, 80), and ferrierrite are about 631, 309, 386, and 224 m^2^/g, respectively. The pore sizes of 13X, ZSM5s (Si/Al = 23, 80), and Ferrierrite using the N_2_ adsorption are 8.07, 6.06, 5.92, and 6.15 Å, respectively. The apparent densities of 13X, ZSM5s (Si/Al = 23, 80), and Ferrierrite are about 1.95 ± 0.04, 1.94 ± 0.01, 1.76 ± 0.08, and 1.92 ± 0.01 g/cm^3^, respectively. The particle sizes of 13X, ZSM5s (Si/Al = 23, 80), and Ferrierrite are 8.26 ± 0.76 μm, 6.24 ± 0.17 μm, 7.08 ± 0.58 μm, and 6.38 ± 0.06 μm, respectively. Figure 2a and Figure 2b show SEM micrographs of doped PPV and NH_4_+ ZSM5(Si/Al = 23) and their mixtures. Zeolite particles are moderately dispersed in the doped PPV matrices.

### 3.3. Ion-Exchange of Zeolites

The ion-exchanged levels of sodium-exchanged zeolites ZSM5 (Si/Al = 23, 80) and Ferrierrite were calculated by taking the Na/Al ratio from XRF results for a theoretical one Al atom to one Na atom [(Na/Al) ×100 = %Na exchange] [6]. The Na ion-exchange levels of ZSM5 (Si/Al = 23, 80) and Ferrierrite are equal to 28%, 17%, and 17%, respectively. In the case of copper-exchanged zeolite ZSM5-23, the theoretical copper-exchange results from two Al atoms for every one Cu^2+^ atom [2(Cu/Al) × 100 = %Cu exchange] [37,38]. The %Cu exchange of ZSM5-23 is about 44%.

### 3.4. Conductivity Measurements

#### 3.4.1. Electrical Conductivity: Steady State Sensitivity to CO

The electrical response (Δσ = σ_CO_-σ_N2before-expose_ [S/cm]) of each sample was calculated from the difference between the saturated conductivity value when exposed to CO and the steady state conductivity value when exposed to pure N_2_ at 1 atm and 30 ± 2 °C. Due to appreciable differences in initial conductivity between various composites, the sensitivity (sensitivity = Δσ/σ_N2_) defined as the electrical conductivity response divided by the electrical conductivity when exposed to pure N_2_ will be used for comparison amongst various materials. Table 2 indicates that the sensitivity values of PPV and doped PPV are 0.06 and 0.54, respectively.

**Table 2:** Electrical conductivity sensitivities and temporal responses of PPV and doped PPV towards CO.

Sample	Electrical conductivity sensitivity (Δσ/σ_N2_)	Response time (t_r_, min)
PPV	6.01E−02	10.26
Doped PPV	5.44E−01	146

**Figure 3:**
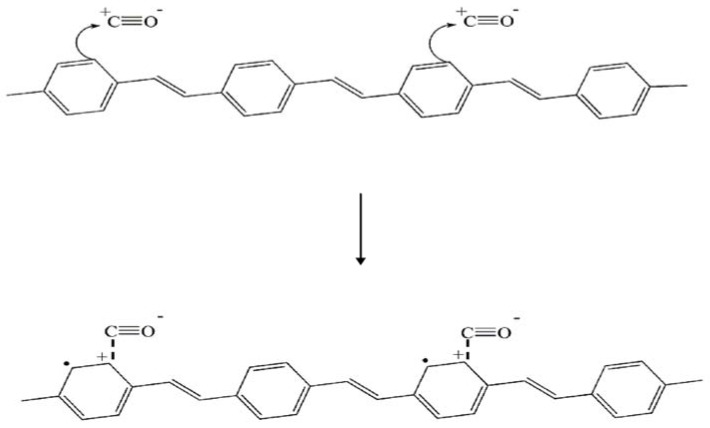
A schematic of the proposed interaction between CO and PPV.

For the undoped PPV, the electrical conductivity response is nearly negligible and the material is rather inert towards CO. The positive increment of the sensitivity of the doped PPV upon exposed to CO can be traced back to the interaction mechanism proposed and shown in Figure 3. CO molecules can act as a secondary dopant, a substance which is subsequently applied to the primary-doped polymer, where charge transfer complex is formed between the polymer and the secondary dopant. This results in a greater number of charges or polarons along the polymer backbone, and hence a larger sensitivity.

Temporal response time (t_r_) is defined as the duration between the starting time of CO exposure to the time at which the conductivity value reaches its steady state. They are 10.26 and 146 sec, for PPV and doped PPV, respectively. The temporal response times are thus indicative of the extent on the interaction between CO and PPV.

#### 3.4.2. Effect of Zeolite Type on Electrical Sensitivity

Three types of zeolite of different pore sizes and Si/Al ratios were used to investigate the effect of zeolite type on the electrical conductivity response to CO. Table 2 lists that the sensitivity values of doped PPV/Na^+^-ZSM5(Si/Al = 23), doped PPV/Na^+^-13X, and doped PPV/Na^+^-Ferrierite; they are 1.48, 1.02, and 0.91, respectively. All composites have higher sensitivity values than that of the pristine PPV and doped PPV, which are 6.01 × 10^–2^ and 5.44 × 10^–1^, respectively. The increase in the sensitivity value of PPV/Zeolite composites relative to that of the pristine PPV and doped PPV suggests that CO molecules are induced and adsorbed into the zeolites through certain interactions. As a result, larger amounts of CO molecules are thus available to interact with PPV.

Doped PPV/Na^+^-ZSM(Si/Al = 23) with the ion-exchanged level equal to 28% has a sensitivity of 1.48, larger than that of doped PPV/Na^+^-13X which is 1.02, even though 13X zeolite contains 100% Na^+^. The higher sensitivity of the doped PPV/Na^+^-ZSM5(Si/Al = 23) may originate from the nature of the zeolite structure itself. Na^+^-ZSM5(Si/Al = 23) zeolite has a channel structure whereas Na-13X zeolite has a cage structure. Nanoporous channel of Na^+^-ZSM5(Si/Al = 23) would allow CO molecules to pass through and to interact with the doped PPV chains more easily relative to that of 13X zeolite.

On the other hand, doped PPV/Na^+^-ZSM5(Si/Al = 23) and doped PPV/Na^+^-FER both have a channel structure with the ion-exchanged levels equal to 28% and 17%, respectively. Na^+^-ZSM5(Si/Al = 23) has a sensitivity value of 1.48, higher than 0.96 of doped PPV/Na^+^-FER. This is simply due to the higher content of Na^+^ which plays an important role as an adsorption site for CO molecules. Na^+^-ZSM5(Si/Al = 23) can induce CO molecules to absorb and thus allows more CO molecules to interact with PPV.

For the effect of Si/Al ratio, two types of zeolite are used to investigate the electrical conductivity response to CO. The sensitivity values of doped PPV/Na^+^-ZSM5(Si/Al = 23) and that of doped PPV/Na^+^-ZSM5(Si/Al = 80) indicate the influence of Na^+^ as in the previous case. The sensitivity value of doped PPV/Na^+^-ZSM5(Si/Al = 23) is 1.48 with the ion-exchanged level of 28%, and that of the doped PPV/Na^+^-ZSM5(Si/Al = 80) is 1.17 with the ion-exchanged level of 17%.

Table 3 shows the temporal response times of the composites studied: doped PPV/Na^+^-13X, the doped PPV/Na^+^-ZSM5(Si/Al = 23), doped PPV/Na^+^-FER, and doped PPV/Na^+^-ZSM5(Si/Al = 80). The temporal induction times are 42, 56, 123, and 153 min, respectively. Therefore, the temporal response time is inversely related to the amount of Na^+^ ion as a first approximation. It appears that a zeolite with more Na^+^ ions can induce CO molecules to absorb faster through the electrostratic interaction, and hence the interaction of CO with PPV chains is terminated in a shorter time.

**Table 3:** Electrical conductivity sensitivities, temporal responses, and ion-exchange levels of PPV/Zeolite composites.

Sample	The sensitivity (∆σ/σ_N2_)	The temporal response (t_r_, min)	Ion-exchanged level (%)
dPPV/13X_Na	1.02E+00	42	100
dPPV/fer_Na	9.16E-01	123	17
dPPV/ZSM5(23)_Na	1.48E+00	56	28
dPPV/ZSM5(80)_Na	1.17E+00	153	17
dPPV/ZSM5(23)_Cu	-1.54E-01	24	44

#### 3.4.3. Effect of Cation Type on Electrical Sensitivity

In this study, the effect of cation type has been investigated using ZSM(Si/Al = 23). In order to minimize the influence of cation size within its framework, Cu^+^ (the radii of the cation is equal to 96 picometers) [42], metal cation with *d* orbital, is chosen to be compared with Na^+^ (the radii of cation is equal to 95 picometers) [42]. The Cu^2+^ ions are 3d^9^ paramagnetic ions and are easily observed by ESR [7]. Cu^+^ ions, on the contrary, are diamagnetic (3d^10^) and therefore are ESR silent [6,7,12]. Our data show that the Cu^2+^ ESR signal of Cu^+^-ZSM5(Si/Al = 23) decreases and the spectral feature changes substantially as a hydrated sample is heated and progressively dehydrated. Therefore, the modification of hydrated system upon dehydrating in vacuum at 573 K undergoes a selfreduction of Cu^2+^ into Cu^+^.

The electrical sensitivity of dPPV/Na^+^-ZSM5(Si/Al = 23) system shows a positive value of 1.48, while the Cu^+^ system give a negative sensitivity of −0.154, as tabulated in Table 2. However, the temporal response time of dPPV/Na^+^-ZSM5(Si/Al = 23) is 53 min, a factor of two greater than that of dPPV/Cu^+^-ZSM5(Si/Al = 23), which is 24 min. To clarify the effect of ion-exchanged types in zeolite on the electrical conductivity sensitivity and the temporal response, a comparison of desorptions of CO has been made on Na^+^-ZSM5(Si/Al = 23) and Cu^+^-ZSM5(Si/Al = 23).

**Figure 4:**
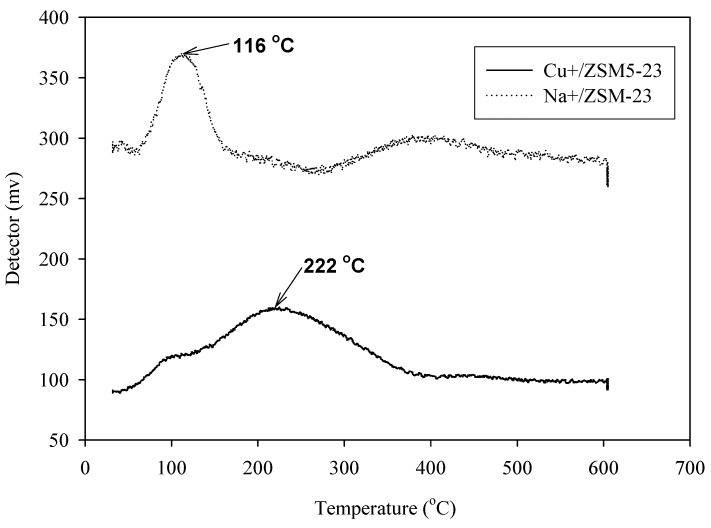
TPD spectra of Na-ZSM5 and Cu-ZSM5 exposed to CO at 298 K and 1 atm.

TPD spectra obtained from the room-temperature adsorption of CO on Na^+^- and Cu^+^-ZSM5(Si/Al = 23) are shown in Figure 4. The temperatures at the maximum desorptions of CO of the two adsorbents are significantly different. For Na^+^-ZSM5(Si/Al = 23), it is observed at 116 °C, while on Cu^+^-ZSM 5(Si/Al = 23) it occurs at 222 °C as shown in Table 4. This result demonstrates that CO molecules are adsorbed more stronger on Cu^+^-ZSM5(Si/Al = 23) than Na^+^-ZSM5(Si/Al = 23). The strong adsorption observed for the copper ion-exchanged ZSM5 sample arises from the interaction between the copper ion and the CO molecule. Carbon monoxide is a weak Lewis base that interacts with coordinatively unsaturated centers, forming pure σ-complexes (in d^0^ systems) and σ, π-complexes (in d*^n^* systems) [11]. The copper ions in ZSM5 act as the active sites for the CO adsorption. In such conditions, it is well known that CO forms stable carbonyls with coordinatively unsaturated Cu^+^ while it does not coordinate with sodium ion. These data clearly confirm the electrical sensitivity and the temporal response results that Cu^+^-ZSM5(Si/Al = 23) is more preferable towards CO molecules than Na^+^-ZSM5(Si/Al = 23). Therefore, a lesser amount of CO molecules are available to interact with the doped PPV chains which causes the negative sensitivity and the faster temporal response.

**Table 4:** The temperatures at the maximum desorptions of CO from Na^+^-ZSM5(Si/Al = 23) and Cu^+^-ZSM 5(Si/Al = 23).

Sample	The temperatures at the maximum desorptions of CO (°C)
Na^+^-ZSM5(Si/Al = 23)	116
Cu^+^-ZSM 5(Si/Al = 23)	222

### 3.5. FTIR Investigation of Interactions of Adsorbed CO

**Figure 5:**
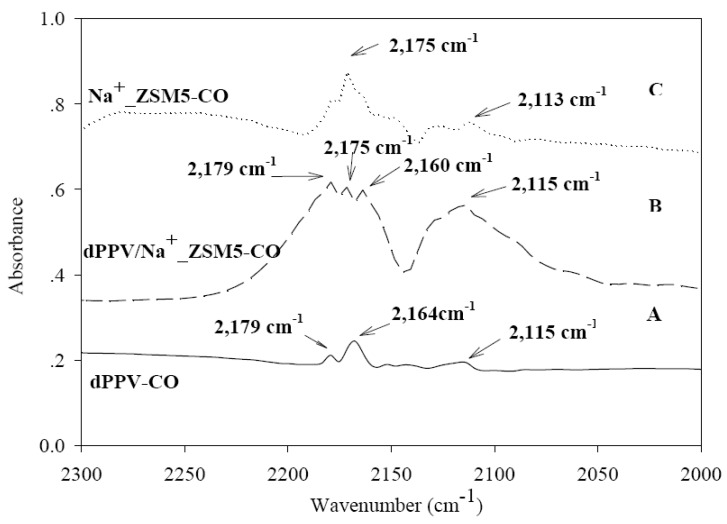
IR spectra of CO (pressure = 1 atm and at room temperature) adsorbed on: (a) doped PPV; (b) doped PPV/Na^+^-ZSM5; and (C) Na^+^-ZSM5.

The interaction of CO with Na-exchanged zeolite and the carbon cation of the quinoid structure of doped PVV can be conveniently monitored by means of IR spectroscopy. The IR spectra in the 2,000–2,300 cm^–1^ region identifying the CO adsorption at 1 atm and at room temperature are shown in Figure 5. The vibrational stretching frequency of free CO molecule (ν = 2,143 cm^–1^) predominately experiences both a blue shift (ν = 2,164 cm^–1^) and a red shift (ν = 2,115 cm^–1^) when CO is physically adsorbed on C^+^ on the quinoid structure of doped PPV (Figure 5A): C^+…^CO and C^+…^OC [39]. In addition, CO molecule may act as a secondary dopant in which charge transfer complex is formed between the polymer and CO molecule: C^+…^CO^–^ as shown in Figure 3. This corresponds to the IR peak at 2,179 cm^–1^.

Figure 5 C shows the IR spectrum of Na^+^_ZSM5-CO. IR peaks at 2,175 cm^–1^ and 2,113 cm^–1^ correspond to the stretching vibrations of physically absorbed CO molecules on the cation of the zeolite through electrostatic interaction. Specifically, the peaks identify Na^+…^CO and Na^+…^OC, respectively. These hysochromic shifts can be readily ascribed to the interactions of CO via the carbon end of its molecule with the positive field set by the cations [8,10,21].

The IR absorption bands for doped PPV/ Na^+^-ZSM5 (Si/Al = 23) appears at 2,175, 2,160, 2,115 and 2,179 cm^–1^: they correspond to Na^+…^CO, C^+…^CO, C^+…^OC and C^+…^CO^–^, respectively. These IR adsorption bands result from the sodium and carbon cation sites present; the first three interactions are electrostatic in nature, and the last is the partially covalent bond through the charge transfer secondary doping on PPV. The band at 2179 cm^–1^ may represent the partially covalent bond C^–…^OC^+^. This band of 2,179 cm^–1^ of the doped PPV/ Na^+^-ZSM5(Si/Al = 23) spectrum have higher intensity than those of the PPV/CO spectrum (Figure 5A). This suggests that the interactions are further induced by the presence of ZSM5(Si/Al = 23). Other significant IR absorptions are also observed; a broad band around 2,110–2,130 cm^–1^ arises from the condensation of CO on the absorbate [40], or from physisorbed CO inside the zeolite structure [41] at high CO equilibrium pressure.

**Figure 6:**
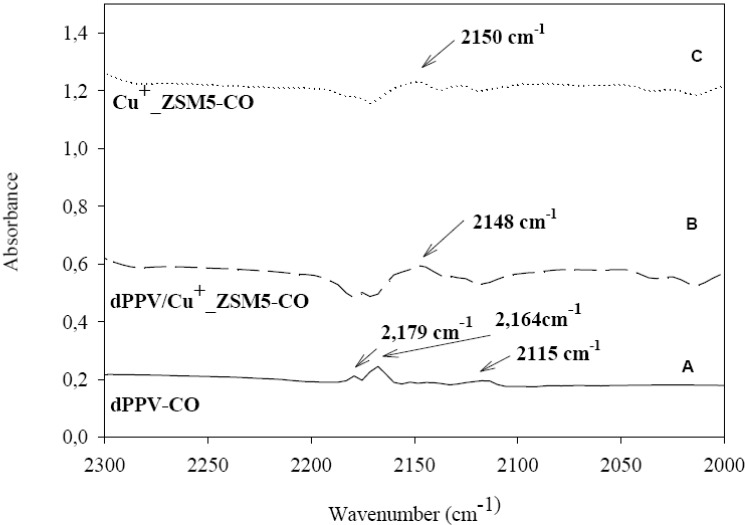
IR spectra of CO (pressure at 1 atm and at room temperature adsorbed on: (a) doped PPV; (b) doped PPV/Cu^+^-ZSM5; and (c) Cu^+^-ZSM5.

Figure 6 C shows the IR spectrum of the absorbed CO species on Cu^+^-ZSM5(Si/Al = 23) at 1 atm and at room temperature. CO is known to be absorbed exceptionally strong on Cu^+^ sites, but only weak on Cu^2+^ [7,14,15,17]. The peak at 2,150 cm^–1^ can be assigned to the adsorption of CO on the Cu-ZSM5, consistent with previous data [42,43,44].

Figure 6 B shows the IR spectrum of the adsorbed CO species on the doped PPV/ Cu^+^-ZSM5 (Si/Al = 23). A smaller peak also occurs at 2,148 cm^–1^; this band is assigned to the interaction between CO^…^Cu^+^. We note that the peaks of 2,179, 2,164 and 2,115 cm^–1^ almost disappear; the interactions between CO and the pristine PPV are nearly diminished. The negative electrical conductivity response of the doped PPV/ Cu^+^-ZSM5(Si/Al = 23) when exposed to CO are due to the disappearances of the peaks at 2,179 and 2,115 cm^–1^ (C^+…^CO and C^+…^OC) and the peak 2,164 cm^−1^ (C^+^---CO^–^). Therefore, it appears that CO molecules are selectively adsorbed on Cu^+^ sites rather than C^+^ sites of the doped PPV.

Finally, we might add that in our previous work we employed various conductive polymers as the matrices and zeolites as the fillers and observed measurable electrical conductivity responses towards toxic gases [45,46,47,48]. In the present work, the composite systems studied consist of the zeolites as the matrices and the conductive PPV as the interacting filler. The latter inverted systems (PPV/Zeolites) are shown to be sensitive to and to react with CO.

## 4. Conclusions

The effects of zeolite type and cation exchanged type on the CO interaction with PPV doped with sulfuric acid were investigated. For the effect of zeolite type, the highest electrical conductivity sensitivity is obtained with the doped PPV/Na^+^-ZSM5(Si/Al = 23); it is slightly higher than those of doped PPV/Na^+^-ZSM5(Si/Al = 80), of the doped PPV/Na^+^-13X, and of the doped PPV/Na^+^-ferrierite. For the effect of sodium ion-exchanged level, the electrical sensitivity values obtained are equal to 1.48, 1.17, 1.02, and 0.91, corresponding to the sodium ion-exchanged levels of 28%, 17%, 100%, and 17%, respectively. Doped PPV/Na^+^-13X possesses the shortest temporal response time, followed by doped PPV/Na^+^-ZSM5(Si/Al = 23), doped PPV/Na^+^-FER, and the doped PPV/Na^+^-ZSM5(Si/Al = 80). The temporal response time is thus inversely related to the amount of Na^+^ ion as a first approximation. For the effect of cation exchanged type, we employed doped PPV/cation exchanged ZSM5 (Si/Al = 23) zeolites (Na^+^ and Cu^+^) as a model. The electrical sensitivity of doped PPV/Cu^+^-ZSM5 system shows the negative value of −0.154, while the Na^+^ system gives a positive sensitivity of 1.48. The IR spectra of the composites indicate a modification of the C-O stretching frequency. Characteristic peaks are: 2,179, 2,175, 2,160 and 2,115 cm^–1^ for doped PPV/Na^+^-ZSM5; 2,148 cm^–1^ for doped PPV/Cu^+^-ZSM5. The TPD results also demonstrate that Cu^+^-ZSM5(Si/Al = 23) adsorbs CO significantly stronger than Na^+^-ZSM5(Si/Al = 23). The stronger adsorption observed for the copper ion-exchange ZSM5 sample arises from the differences in the interactions between CO and the sodium ion-exchanged ZSM5 through electrostatic interaction, and CO and the copper ion-exchanged ZSM5 through electrostatic interaction and a complex formation. The copper exchanged ZSM5 allows a lesser amount of CO molecules to interact with the doped PPV chains and the complex formed reduces electron mobility are the primary causes for the negative sensitivity value and the faster temporal response observed.
